# Supplementary material for: Simply cut out – Combining CRISPR/Cas9 RNPs and transiently selected telomere vectors for marker free-gene deletion in Trichoderma atroviride
Source: Front Genome Ed. 2025 Jul 2;7:1623963. doi: 10.3389/fgeed.2025.1623963 (PMC12263576; doi:10.3389/fgeed.2025.1623963)
Supplement: Supplementary file 1 [file Presentation1.pptx]

## Slide 1
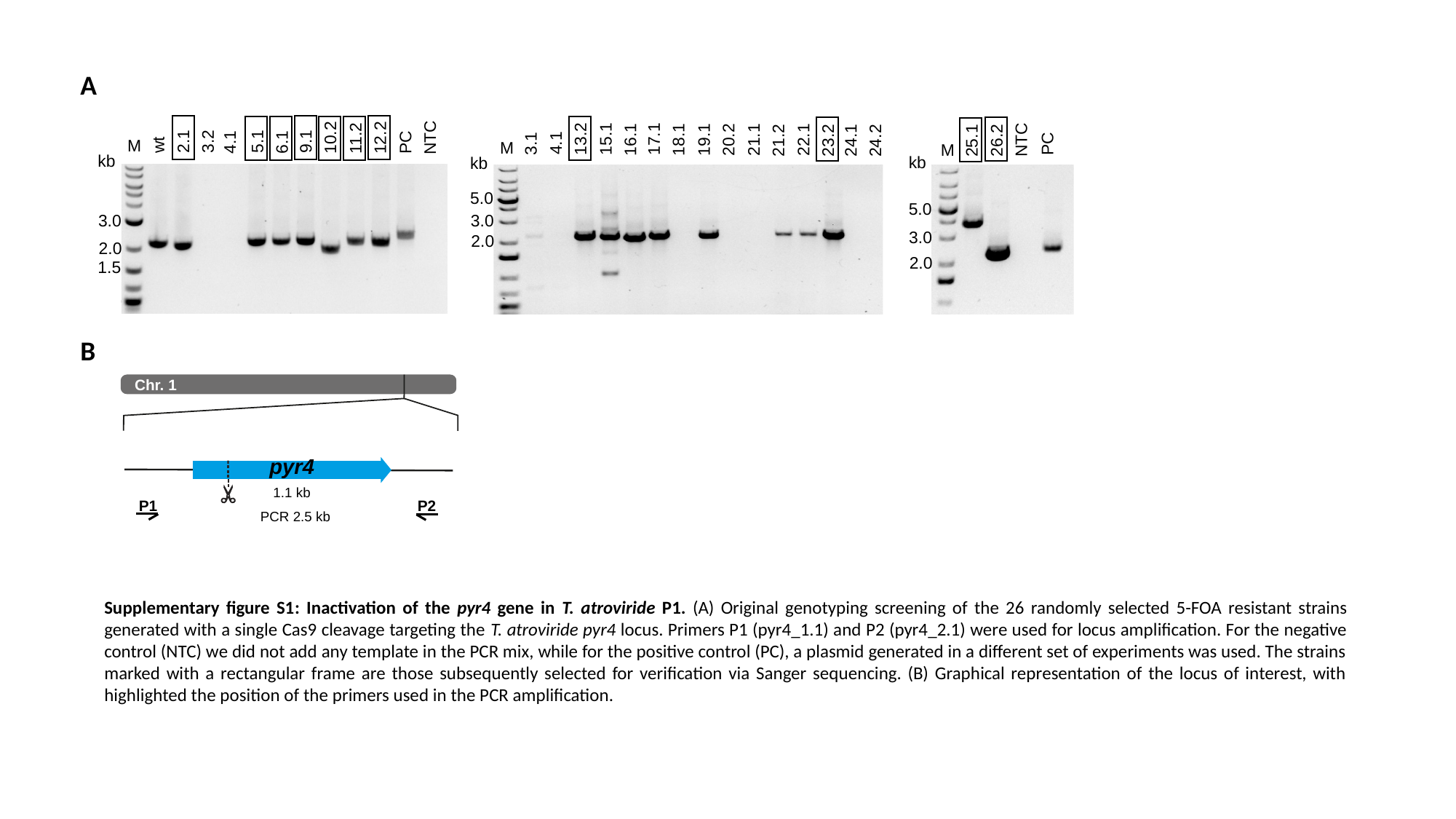

A
15.1
16.1
PC
10.2
11.2
12.2
PC
NTC
17.1
18.1
19.1
20.2
21.1
22.1
21.2
23.2
24.1
24.2
26.2
13.2
25.1
NTC
2.1
wt
3.2
5.1
9.1
4.1
6.1
4.1
3.1
M
M
M
kb
kb
kb
5.0
5.0
3.0
3.0
3.0
2.0
2.0
2.0
1.5
B
Chr. 1
pyr4
1.1 kb
P1
P2
PCR 2.5 kb
Supplementary figure S1: Inactivation of the pyr4 gene in T. atroviride P1. (A) Original genotyping screening of the 26 randomly selected 5-FOA resistant strains generated with a single Cas9 cleavage targeting the T. atroviride pyr4 locus. Primers P1 (pyr4_1.1) and P2 (pyr4_2.1) were used for locus amplification. For the negative control (NTC) we did not add any template in the PCR mix, while for the positive control (PC), a plasmid generated in a different set of experiments was used. The strains marked with a rectangular frame are those subsequently selected for verification via Sanger sequencing. (B) Graphical representation of the locus of interest, with highlighted the position of the primers used in the PCR amplification.

## Slide 2
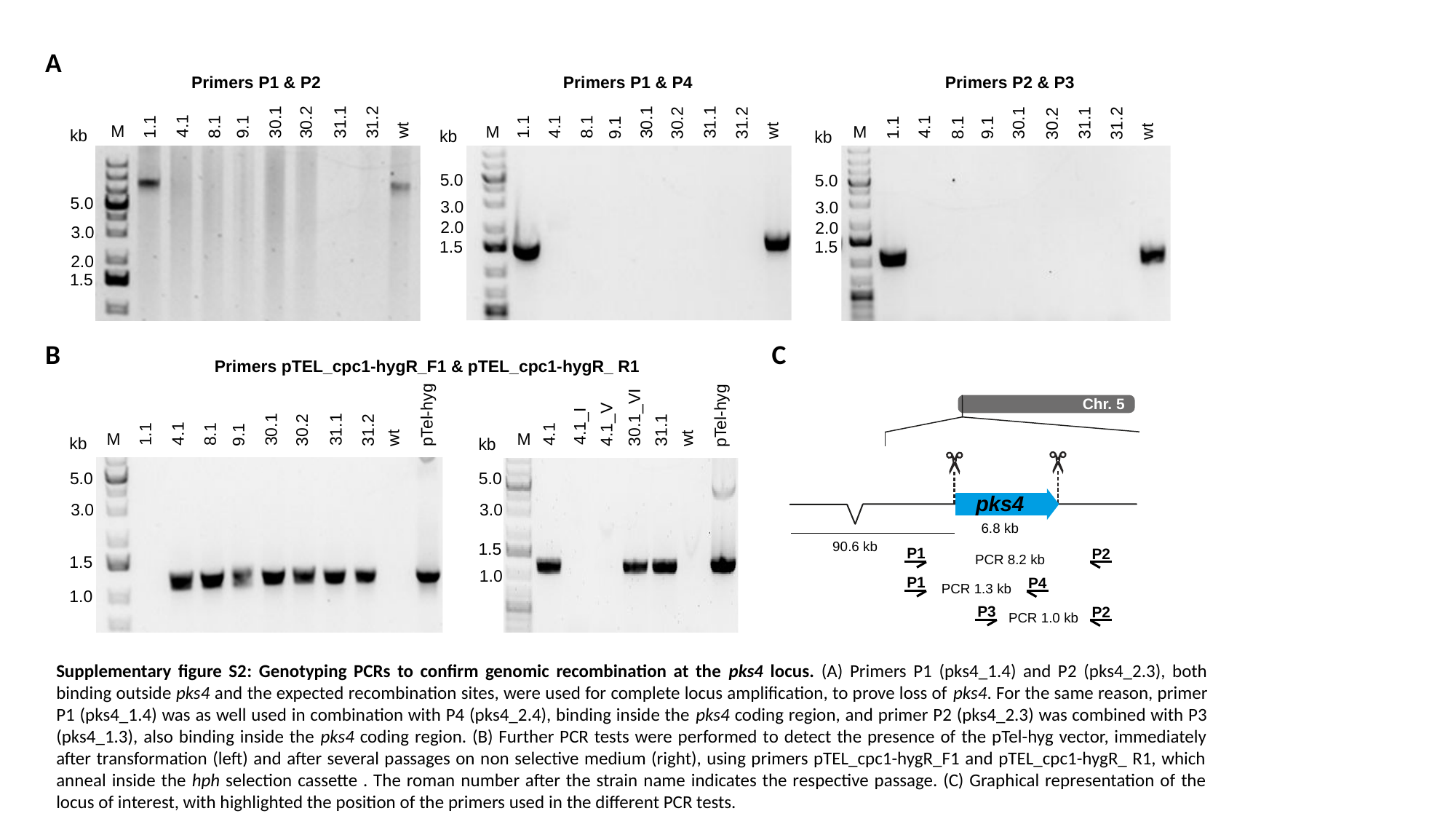

A
Primers P1 & P2
Primers P1 & P4
Primers P2 & P3
30.1
31.1
31.2
wt
30.2
30.1
31.1
31.2
wt
30.2
30.1
31.1
31.2
wt
30.2
4.1
1.1
8.1
4.1
9.1
1.1
8.1
4.1
9.1
1.1
8.1
9.1
M
M
M
kb
kb
kb
5.0
5.0
5.0
3.0
3.0
2.0
2.0
3.0
1.5
1.5
2.0
1.5
C
B
Primers pTEL_cpc1-hygR_F1 & pTEL_cpc1-hygR_ R1
Chr. 5
pks4
6.8 kb
90.6 kb
30.1_VI
pTel-hyg
pTel-hyg
4.1
4.1_I
4.1_V
30.1
31.1
31.2
wt
30.2
31.1
wt
4.1
1.1
8.1
9.1
M
M
kb
kb
5.0
5.0
3.0
3.0
1.5
P1
P2
PCR 8.2 kb
1.5
1.0
P1
P4
PCR 1.3 kb
1.0
P3
P2
PCR 1.0 kb
Supplementary figure S2: Genotyping PCRs to confirm genomic recombination at the pks4 locus. (A) Primers P1 (pks4_1.4) and P2 (pks4_2.3), both binding outside pks4 and the expected recombination sites, were used for complete locus amplification, to prove loss of pks4. For the same reason, primer P1 (pks4_1.4) was as well used in combination with P4 (pks4_2.4), binding inside the pks4 coding region, and primer P2 (pks4_2.3) was combined with P3 (pks4_1.3), also binding inside the pks4 coding region. (B) Further PCR tests were performed to detect the presence of the pTel-hyg vector, immediately after transformation (left) and after several passages on non selective medium (right), using primers pTEL_cpc1-hygR_F1 and pTEL_cpc1-hygR_ R1, which anneal inside the hph selection cassette . The roman number after the strain name indicates the respective passage. (C) Graphical representation of the locus of interest, with highlighted the position of the primers used in the different PCR tests.

## Slide 3
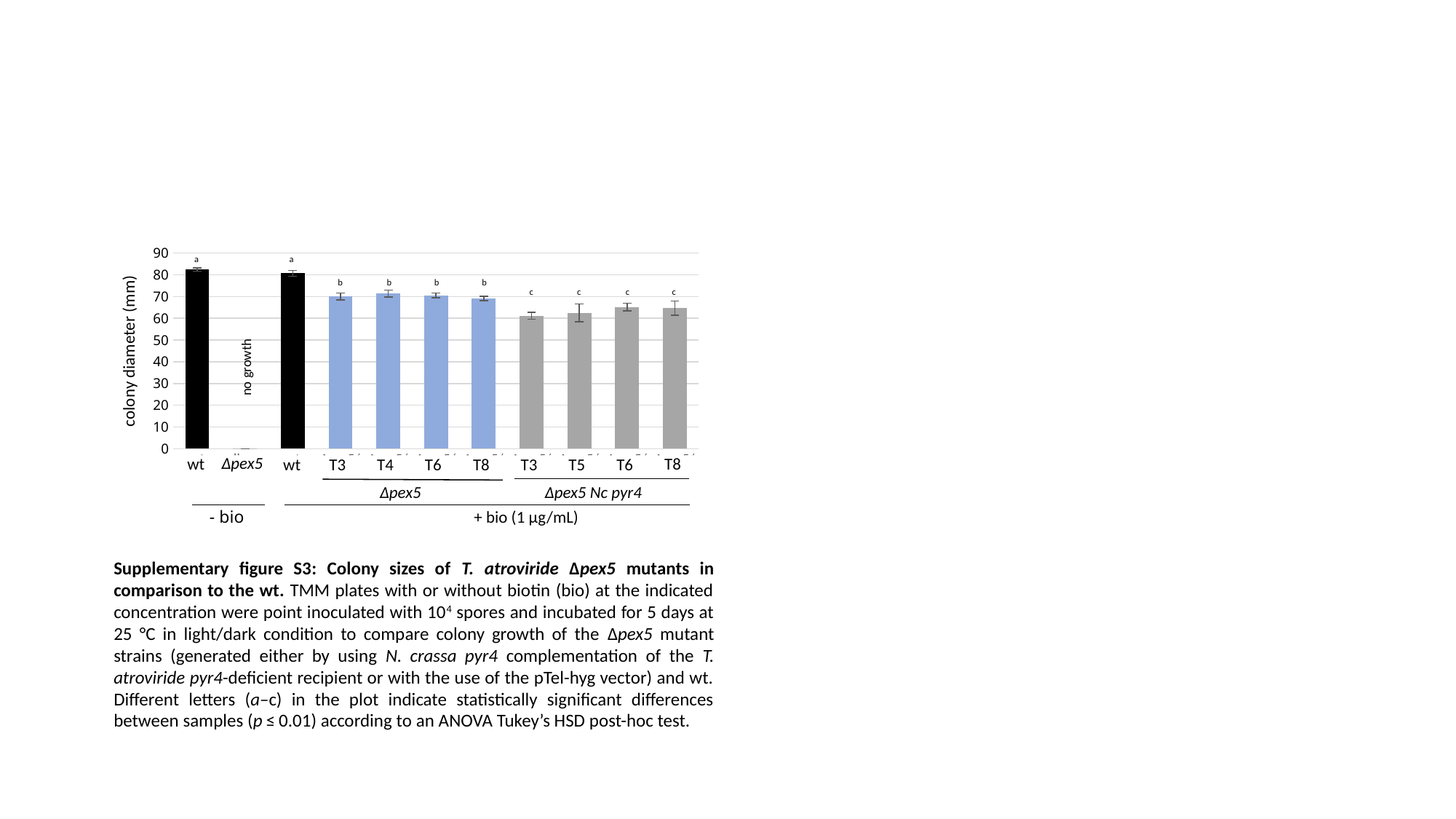

### Chart
| Category | |
|---|---|
| wt | 82.5 |
| all pex | 0.0 |
| wt | 80.66666666666667 |
| ∆pex5/ pTEL T6.1 | 70.0 |
| ∆pex5/ pTEL T11.1 | 71.33333333333333 |
| ∆pex5/ pTEL T14.1 | 70.5 |
| ∆pex5/ pTEL T19.1 | 69.16666666666667 |
| ∆pex5/ pyr4 T5.1 | 61.166666666666664 |
| ∆pex5/ pyr4 T10.1 | 62.5 |
| ∆pex5/ pyr4 T11.1 | 65.16666666666667 |
| ∆pex5/ pyr4 T15.1 | 64.66666666666667 |a
a
b
b
b
b
c
c
c
c
colony diameter (mm)
 no growth
wt
T8
wt
T3
T4
T6
T8
T3
T5
T6
∆pex5
∆pex5 Nc pyr4
+ bio (1 µg/mL)
∆pex5
- bio
Supplementary figure S3: Colony sizes of T. atroviride Δpex5 mutants in comparison to the wt. TMM plates with or without biotin (bio) at the indicated concentration were point inoculated with 104 spores and incubated for 5 days at 25 °C in light/dark condition to compare colony growth of the Δpex5 mutant strains (generated either by using N. crassa pyr4 complementation of the T. atroviride pyr4-deficient recipient or with the use of the pTel-hyg vector) and wt. Different letters (a–c) in the plot indicate statistically significant differences between samples (p ≤ 0.01) according to an ANOVA Tukey’s HSD post-hoc test.
